# YQFC: a web tool to compare quantitative biological features between two yeast gene lists

**DOI:** 10.1093/database/baaa076

**Published:** 2020-11-11

**Authors:** Wei-Sheng Wu, Lai-Ji Wang, Han-Chen Yen, Yan-Yuan Tseng

**Affiliations:** Department of Electrical Engineering, National Cheng Kung University, No.1, University Road, Tainan city, 70101 Taiwan; Department of Electrical Engineering, National Cheng Kung University, No.1, University Road, Tainan city, 70101 Taiwan; Department of Electrical Engineering, National Cheng Kung University, No.1, University Road, Tainan city, 70101 Taiwan; Center for Molecular Medicine and Genetics, Wayne State University, School of Medicine, 3127 Scott Hall, 540 East Canfield, Detroit, MI 48201, USA

## Abstract

Nowadays high-throughput omics technologies are routinely used in biological research. From the omics data, researchers can easily get two gene lists (e.g. stress-induced genes vs. stress-repressed genes) related to their biological question. The next step would be to apply enrichment analysis tools to identify distinct functional/regulatory features between these two gene lists for further investigation. Although various enrichment analysis tools are already available, two challenges remain to be addressed. First, most existing tools are designed to analyze only one gene list, so they cannot directly compare two gene lists. Second, almost all existing tools focus on identifying the enriched qualitative features (e.g. gene ontology [GO] terms, pathways, domains, etc.). Many quantitative features (e.g. number of mRNA isoforms of a gene, mRNA half-life, protein half-life, transcriptional plasticity, translational efficiency, etc.) are available in the yeast, but no existing tools provide analyses on these quantitative features. To address these two challenges, here we present Yeast Quantitative Features Comparator (YQFC) that can directly compare various quantitative features between two yeast gene lists. In YQFC, we comprehensively collected and processed 85 quantitative features from the yeast literature and yeast databases. For each quantitative feature, YQFC provides three statistical tests (t-test, U test and KS test) to test whether this quantitative feature is statistically different between the two input yeast gene lists. The distinct quantitative features identified by YQFC may help researchers to study the underlying molecular mechanisms that differentiate the two input yeast gene lists. We believe that YQFC is a useful tool to expedite the biological research that uses high-throughput omics technologies.

**Database URL**: http://cosbi2.ee.ncku.edu.tw/YQFC/

## Introduction

Nowadays high-throughput omics technologies (e.g. genomics, transcriptomics, proteomics, etc.) are routinely used in biological research (1). From the omics data, researchers can easily get two gene lists (e.g. stress-induced genes vs. stress-repressed genes, drug-induced genes vs. drug-repressed genes, hub genes vs. nonhub genes, genes with long mRNA half-life vs. genes with short mRNA half-life, etc.) related to their biological question. The ultimate goal is to understand the underlying molecular mechanisms that differentiate these two gene lists. To achieve this goal, researchers need to know which biological features are good candidates for further investigation. Since enrichment analysis tools can help researchers to identify possible biological features, which are worthy of pursue, the enrichment analysis tool development has become a popular research topic in the bioinformatics field (2).

Many enrichment analysis tools are available for analyzing yeast gene lists. First, DAVID (3) provides gene ontology enrichment, pathway enrichment, protein domain enrichment and chromosome enrichment analyses. Second, YeastMine (4) provides gene ontology enrichment, pathway enrichment and publication enrichment analysis. Third, modPhEA (5) provides phenotype enrichment analysis. Fourth, YGA (6) provides physical/genetic interaction enrichment and functional group enrichment analyses. Fifth, YHMI (7) provides histone modification enrichment analysis. Sixth, YARG (8) provides arsenic-related genes enrichment analysis. Seventh, YEASTRACT (9) provides single transcription factor regulation enrichment analysis. Eighth, YCRD (10) provides cooperative transcription factor pair regulation enrichment analysis. Descriptions of many other enrichment analysis tools could be found in a review paper (2).

Although many enrichment analysis tools are already available for testing various biological features, two challenges remain to be addressed. First, most existing tools are designed to analyze one given gene list, so they (e.g. YeastMine, YHMI, YARG, YEASTRACT, YCRD, etc.) cannot directly compare two given gene lists. Second, almost all existing tools (e.g. YeastMine, DAVID, modPhEA, YHMI, etc.) focus on finding the enriched qualitative features (e.g. gene ontology [GO] terms, pathways, domains, phenotypes, histone modifications, etc.). Many quantitative features (e.g. mRNA expression level, mRNA half-life, transcriptional plasticity, translational efficiency, protein abundance, protein half-life, UTR length, number of mRNA isoforms of a gene, etc.) are available in the yeast, but no existing tools provide analyses on these quantitative features. To address these two challenges, here we develop Yeast Quantitative Features Comparator (YQFC) to directly compare various quantitative features between two yeast gene lists.

In YQFC, we comprehensively collected and processed 85 quantitative features from the yeast literature and yeast databases. For each quantitative feature, YQFC provides three statistical tests (t-test, U test and KS test) to test whether this quantitative feature is statistically different between the two given yeast gene lists. Both tables and figures (box plots and cumulative distribution function [CDF] plots) are provided to visualize the testing results.

## Construction and contents

### Collection of 85 quantitative features from 7 yeast publications and 6 yeast databases

We comprehensively collected and processed 85 quantitative features from 7 yeast publications (11–17) and 6 yeast databases (YeastMine, YeastNet (18), YAGM (19), SGD (20), BioGRID (21), SPELL (22)). We classified these 85 quantitative features into 4 categories: 12 gene features, 4 mRNA features, 52 protein features, and 17 network features (Table 1). The detailed source information of all quantitative features in YQFC is given in Supplementary Table 1.

**Table 1.** The 85 collected quantitative features could be divided into 4 categories: 12 gene features, 4 mRNA features, 52 protein features and 17 network features

12 Gene features	CDS length, 5ʹUTR length, 3ʹUTR length, Number of publications, number of GO terms, number of GO slim terms, number of pathways, number of mutant phenotypes, number of mRNA isoforms, number of transcriptional regulators, number of fungal homologs, number of nonfungal and *Saccharomyces cerevisiae* homologs
4 mRNA features	mRNA level (3 datasets^a^), mRNA half-life, transcriptional plasticity, translational efficiency (5 datasets^b^)
52 protein features	Number of domains, number of PTMs, protein half-life, amino acid composition (20 kinds^c^), atomic composition (5 kinds^d^), extinction coefficient at 280 nm (2 kinds^e^), protein abundance (in the normal growth condition, 23 datasets^f^), protein abundance (in various stress conditions, 11 kinds^g^), protein physical details (protein length, molecular weight, pI, aliphatic index, instability index), coding region translation calculation (codon bias, codon adaptation index, frequency of optimal codons, hydropathicity of protein, aromaticity score)
17 network features	Number of interactors in the following networks:
	PI (3 datasets^h^) network GI network CC network CX network DC network GN (similar genomic context) network GT (similar profiles of GI partners) network PG (similar phylogenetic profiles) network TS (3-D protein structure of interacting orthologous proteins) network EPA network FAA network GIA network LEA network MPA network PIA network TFBA network TFRA network

CC, co-citation; CDS, coding sequence; CX, co-expression; DC, domain co-occurrence; EPA, expression profile association; FAA, functional annotation association; GI, genetic interaction; GIA, genetic interaction association; LEA, literature evidence association; MPA, mutant phenotype association; pI, isoelectric point; PI, physical interaction; PIA, physical interaction association; PTM, posttranslational modification; TFBA, transcription factor-binding association; TFRA, transcription factor regulation association; UTR, untranslated region.

a We collected three datasets of mRNA levels generated from microarray (11) and RNA-seq (12).

b We downloaded five datasets of translational efficiency from Csárdi *et al.* (16) who collected them from the literature.

c Amino acid composition contains 20 kinds of amino acids.

d Atomic composition contains five kinds of atoms: carbon, hydrogen, nitrogen, oxygen, and sulfur.

e Extinction coefficient at 280 nm contains two kinds: (i) all cys residues appear as half cystines and (ii) no cys residues appear as half cystines.

f We downloaded 23 datasets of protein abundance measured in the normal growth condition from Ho *et al.* (17) who collected them from the literature.

g We downloaded the datasets of protein abundance measured in 11 different stress conditions (dithiothretiol, H_2_O_2_, hydroxyurea, methyl methanesulfonate, etc.) from Ho *et al.* (17) who collected them from the literature.

h We collected three datasets of protein-protein physical interaction from BioGRID (21) and YeastNet (18).

PI (physical interaction, 3 datasets^h^) network

GI (genetic interaction) network

CC (co-citation) network

CX (co-expression) network

DC (domain co-occurrence) network

GN (similar genomic context) network

GT (similar profiles of genetic interaction partners) network

PG (similar phylogenetic profiles) network

TS (3-D protein structure of interacting orthologous proteins) network

EPA (expression profile association) network

FAA (functional annotation association) network

GIA (genetic interaction association) network

LEA (literature evidence association) network

MPA (mutant phenotype association) network

PIA (physical interaction association) network

TFBA (transcription factor binding association) network

TFRA (transcription factor regulation association) network

YQFC collected and processed data of 85 quantitative features in yeast. For each yeast gene, the values of all these 85 quantitative features are stored. Users can download our unique and useful feature data from the Download page of YQFC.

### Three statistical tests (t-test, U test and KS test) used to compare a quantitative feature between two yeast gene lists

For each quantitative feature, YQFC provides three statistical tests (t-test, U test and KS test) to test whether this quantitative feature is statistically different between the two given yeast gene lists (denoted as *L1* and *L2*). For example, assume that a user wants to compare the 5ʹUTR length between *L1* and *L2*. YQFC provides the following three statistical tests to do this task. First, the t-test (or called Student’s t-test) is used to determine if the mean 5ʹUTR length of the genes in *L1* is statistically longer/shorter than the mean 5ʹUTR length of the genes in *L2* (23). Note that the t-test is a parametric test based on the assumption that the observed data come from normal distributions. Second, the U test (or called Mann-Whitney U test) is used to determine if the median 5ʹUTR length of the genes in *L1* is statistically longer/shorter than the median 5′UTR length of the genes in *L2* under the location shift assumption (24). Note that the U test is a nonparametric test that does not assume anything about the distribution of the underlying populations. Third, the KS test (or called Kolmogorov-Smirnov test) is used to determine if the cumulative distribution of the 5′UTR length of the genes in *L1* is statistically larger/smaller than the cumulative distribution of the 5′UTR length of the genes in *L2* (24). Note that the KS test is a nonparametric test that does not assume anything about the distribution of the underlying populations.

### Implementation of the web interface of YQFC

**Figure 1.**
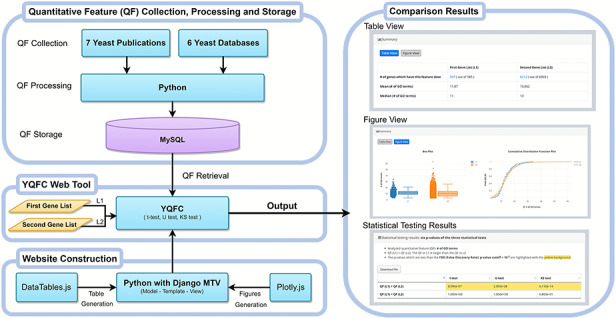
The configuration of YQFC.

Figure 1 illustrates the configuration of YQFC. The web interface of YQFC was developed in Python using the Django MTV framework. The 85 processed quantitative feature data were deposited in MySQL. All tables, box plots, and CDF plots were produced by the JavaScript and feature-rich JavaScript libraries (jQuery, DataTables and Plotly.js) to visualize data on the webpage. Except for the main website (http://cosbi2.ee.ncku.edu.tw/YQFC/), we also have two backup sites (http://cosbi4.ee.ncku.edu.tw/YQFC/ and http://cosbi6.ee.ncku.edu.tw/YQFC/).

## Utility and discussion

### The usage of YQFC

**Figure 2.**
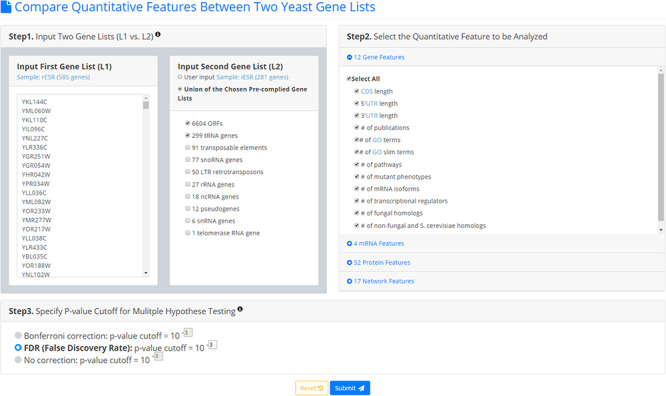
The input page. To use YQFC, users have to go through a three-step process.

YQFC is a web tool for identifying the distinct quantitative features between two input yeast gene lists. To use YQFC, users have to go through a three-step process (Figure 2). [Step 1] Users need to input two gene lists to be compared. Standard names, systematic names, or aliases are all acceptable. If users only have one input gene list, they can use our precomplied gene lists (e.g. 6604 ORF genes, 299 tRNA genes, 27 rRNA genes, etc.) to serve as the second input gene list. In other words, YQFC also supports analysis of a single gene list. [Step 2] Users need to select the quantitative features to be analyzed. [Step 3] Since YQFC tests many quantitative features (i.e. multiple hypotheses testing), users have to select a statistical method (Bonferroni correction or false discovery rate (FDR)) for multiple hypotheses correction and set the *P*-value threshold. Note that using different correction method will give you different corrected *P*-value. Bonferroni correction is more conservative than FDR That is, Bonferroni correction has a smaller type I error rate, resulting in a smaller power, than FDR does. The *P*-value threshold determines the statistical significance of how different of a quantitative feature is between the two input gene lists. The more stringent the *P*-value threshold, the higher the statistical significance of the identified distinct quantitative feature.

**Figure 3.**
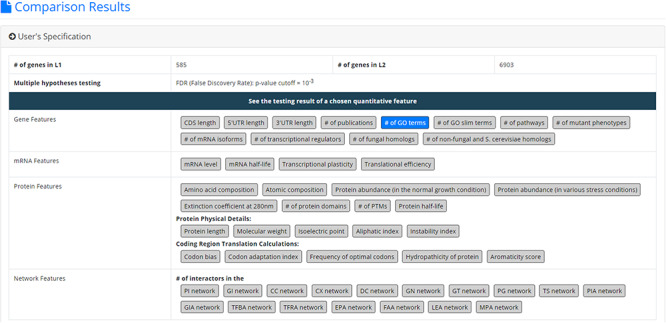
The result page (the first part). The first part contains the information of the user’s settings (i.e. the number of genes in *L1*, the number of genes in *L2*, the selected quantitative features, the selected multiple hypotheses correction method, and the selected p-value threshold).

**Figure 4.**
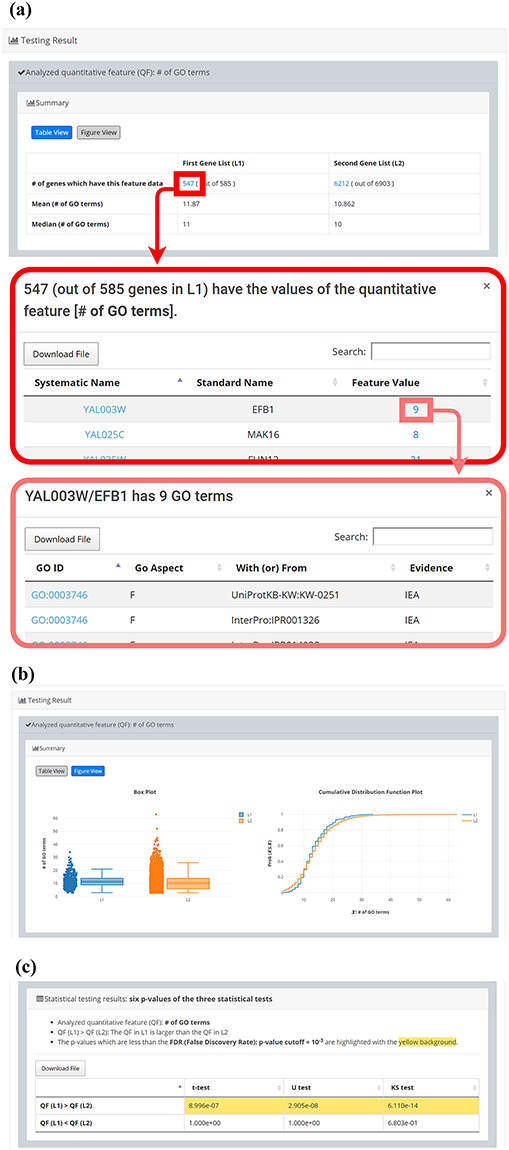
The result page (the second part). The second part contains the result of each selected quantitative feature shown as two sections: (i) Summary and (ii) Statistical testing results. In the “Summary” section, users can choose “Table View” or “Figure View”. (a) “Table View” provides a table containing two kinds of information. First, the numbers of genes (in *L1* and *L2*, respectively) which have the feature values are given. If users click on the number, they will see the names and the feature values of these genes. By clicking on a feature value, users will see the original sources of the feature value. Second, the mean and median feature values of the genes (having feature values) in *L1* and *L2*, respectively, are given. (b) “Figure View” provides two kinds of plots for visualization. Box plots are used to display variation in the feature values in *L1* and *L2*, respectively. Cumulative distribution function (CDF) plots are used to show the probability that the feature value X is less than or equal to a specific value x (i.e. Prob(X$\leq$x)). (c) In the “Statistical testing results” section, users can see a table with six *p*-values. Three *p*-values (calculated by t-test, U test, and KS test) represent the statistical significance of claiming the quantitative feature of *L1* is larger than that of *L2* (denoted as QF(*L1*)>QF(*L2*)). The other three *p*-values (calculated by t-test, U test, and KS test) represent the statistical significance of claiming the quantitative feature of *L1* is smaller than that of *L2* (denoted as QF(*L1*)<QF(*L2*)). To draw the users’ attention, the *p*-values which are less than the *p*-value threshold are highlighted with the yellow background.

After submission, YQFC will perform three statistical tests (t-test, U test and KS test) to test whether a selected quantitative feature is statistically different between the two input gene lists (denoted as *L1* and *L2*). This process will go through all the selected quantitative features. Once the analysis process is complete, YQFC will return the results with two parts. The first part (Figure 3) contains the information of the user’s settings (i.e. the number of genes in *L1*, the number of genes in *L2*, the selected quantitative features, the selected multiple hypotheses correction method, and the selected *P*-value threshold). The second part contains the result of each selected quantitative feature shown as two sections: (i) Summary and (ii) Statistical testing results. In the ‘Summary’ section, users can choose ‘Table View’ or ‘Figure View’. ‘Table View’ (Figure 4a) provides a table containing two kinds of information. First, the numbers of genes (in *L1* and *L2*, respectively), which have the feature values are given. If users click on the number, they will see the names and the feature values of these genes. By clicking on a feature value, users will see the original sources of the feature value. Second, the mean and median feature values of the genes (having feature values) in *L1* and *L2*, respectively, are given. ‘Figure View’ (Figure 4b) provides two kinds of plots for visualization. Box plots are used to display variation in the feature values in *L1* and *L2*, respectively. CDF plots are used to show the probability that the feature value $X$ is less than or equal to a specific value $x$ (i.e. $Prob\left( {X \le x} \right)$). In the ‘Statistical testing results’ section (Figure 4c), users can see a table with six *P*-values. Three *P*-values (calculated by t-test, U test and KS test) represent the statistical significance of claiming the quantitative feature of *L1* is larger than that of *L2* (denoted as $QF\left( {{L_1}} \right) \gt QF\left( {{L_2}} \right)$). The other three *P*-values (calculated by t-test, U test and KS test) represent the statistical significance of claiming the quantitative feature of *L1* is smaller than that of *L2* (denoted as $ QF\left( {{L_1}} \right) \lt QF\left( {{L_2}} \right)$). To draw the users’ attention, the *P*-values which are less than the *P*-value threshold are highlighted with the yellow background. To ensure the robustness of the testing results, we suggest the users to investigate the quantitative features with statistical significance in all three statistical tests (t-test, U test and KS test). As for the *P*-value threshold, we suggest the users to use 0.01 or less. The more stringent the *P*-value threshold, the less testing results that can be called statistical significance.

### A case study

Microarray experiments have identified approximately 900 environmental stress response (ESR) genes whose mRNA levels respond to various environmental stresses (e.g. heat shock, oxidative stress, osmotic stress, etc.) in a stereotypical manner (25). These ESR genes can be divided into 585 repressed ESR (rESR) genes and 281 induced ESR (iESR) genes. Previous studies have shown that many rESR genes are housekeeping genes (e.g. ribosomal genes and genes involved in growth-related processes), while iESR genes are usually involved in various stress defense mechanisms (6, 25). We are interested in knowing which quantitative features can differentiate rESR genes from iESR genes. Therefore, we input these two gene lists (first list: 585 rESR genes and second list: 281 iESR genes) into our YQFC as a case study (Supplementary Table 2).

Many interesting observations have revealed from the analysis results of our YQFC. To ensure the statistical significance and robustness of the observations, we only discuss those with FDR-corrected *P*-values < 0.001 in all three statistical tests (t-test, U test and KS test). First, in the normal growth condition, rESR genes have higher mRNA level, larger protein abundance and larger translational efficiency than iESR genes do (Table 2). These observations are not surprising. Since many rESR genes are housekeeping genes, their proteins are highly needed in the normal growth condition. On the contrary, iESR genes are involved in the stress defense mechanisms, so their proteins are not highly needed in the normal growth condition. Second, rESR genes also have larger protein abundance than iESR genes do (Table 2) in various stress conditions (e.g. dithiothretiol, H_2_O_2_, hydroxyurea, and methyl methanesulfonate). This indicates that even in the stress conditions, the needed quantity of housekeeping proteins are still larger than that of the stress response proteins to maintain the cell physiology. Third, in the normal growth condition, rESR genes have shorter mRNA half-life than iESR genes do (Table 3), consistent with a previous study reporting that ribosomal genes (belonging to rESR genes) have short mRNA half-life (26). Fourth, iESR genes have larger transcriptional plasticity, more transcriptional regulators and longer 5ʹUTR length than rESR genes do (Table 3). Since iESR proteins are only highly needed in the stress conditions, their expression must be under complicated controls. Having larger transcriptional plasticity and more transcriptional regulators indicate that the transcription of iESR genes may be more tightly regulated than that of rESR genes. Our finding is supported by a previous study reporting that the stress-induced genes are under a more complicated transcriptional regulation than the housekeeping genes (27). Since 5ʹUTR is known to contain translational regulation signals (28), having longer 5′UTR suggests that the translation of iESR genes may be under more complicated regulation than that of rESR genes. For example, uORFs are one kind of translational regulation signals (29). It is known that the proportion of genes that contain uORFs in the 5ʹUTR is much greater for translationally up-regulated genes than down-regulated genes under various stress conditions (29). Therefore, we suspect that this observation is also hold for iESR genes vs. rESR genes. Indeed, we found that the proportion of genes that contain uORFs in the 5ʹUTR is much greater for iESR genes than rESR genes (Supplementary Table 3). Fifth, rESR genes have more GO term annotations, higher mRNA isoforms, and more interactors in the physical/genetic networks than iESR genes do. The biological meaning of these observations still needs further investigation. Several other quantitative features that can differentiate rESR genes from iESR genes could be found in Tables 2 and 3.

**Table 2.** YQFC found that rESR genes are statistically larger than iESR genes in 23 QFs. To ensure the statistical significance and robustness of the findings, we only consider those with FDR-corrected *P*-values < 0.001 in all three statistical tests (t-test, U test and KS test)

QF(rESR) > QF(iESR)	*P*-value (t-test)	*P*-value (U test)	*P*-value (KS test)
Number of mRNA isoforms	2.393E-36	1.047E-33	1.421E-24
Number of GO terms	1.074E-04	2.751E-06	1.341E-06
mRNA level—mRNA expression level	1.925E-37	1.390E-53	3.282E-44
Translational efficiency—Dataset1 (Csárdi)	3.143E-17	1.955E-29	7.422E-27
Protein abundance (normal condition)—Dataset1 (Mean molecules per cell)	3.597E-11	2.328E-23	1.580E-18
Protein abundance (stress condition)—2 mM dithiothreitol, 2 h	8.394E-05	7.610E-07	9.150E-05
Protein abundance (stress-condition)—1mM H_2_O_2_, 1 h	3.487E-07	6.236E-13	5.831E-09
Protein abundance (stress-condition)—0.2M hydroxyurea, 160 min	1.193E-11	3.315E-16	1.748E-10
Protein abundance (stress-condition)—0.2M hydroxyurea, 2 h	3.708E-08	1.920E-10	1.715E-08
Protein abundance (stress-condition)—0.03% methyl methanesulfonate, 2 h	3.371E-06	2.133E-10	2.027E-09
Codon bias	9.216E-38	7.312E-21	2.745E-16
Codon adaptation index	1.313E-45	1.472E-28	6.607E-21
Frequency of optimal codons	3.524E-38	2.1344E-21	2.180E-16
Atomic composition—Hydrogen	3.4931E-16	1.449E-15	8.345E-12
Atomic composition—Nitrogen	1.134E-06	7.026E-07	1.582E-05
Amino acid composition—A	7.937E-07	2.9351E-07	5.746E-06
Amino acid composition—E	3.997E-06	7.183E-05	1.094E-05
Amino acid composition—K	2.466E-15	1.033E-11	8.844E-12
Amino acid composition—R	2.879E-16	6.514E-11	9.999E-10
Amino acid composition—V	3.859E-07	2.224E-06	1.899E-04
Number of interactors in the PI network—BioGRID	1.123E-14	2.408E-41	2.631E-34
Number of interactors in the GI network	3.556E-19	1.530E-18	1.226E-19
Number of interactors in the CX network	3.079E-07	1.900E-05	6.763E-06

QFs quantitative features

**Table 3.** YQFC found that iESR genes are statistically larger than rESR genes in 17 QFs. To ensure the statistical significance and robustness of the findings, we only consider those with FDR-corrected *P*-values < 0.001 in all three statistical tests (t-test, U test and KS test)

QF(iESR) > QF(rESR)	*P*-value (t test)	*P*-value (U test)	*P*-value (KS test)
5ʹUTR length	9.172E-07	1.932E-14	2.831E-12
Number of transcriptional regulators	1.544E-06	6.367E-06	7.281E-04
mRNA half-life	3.335E-32	6.538E-78	3.760E-71
Transcriptional plasticity	1.747E-07	4.046E-08	6.679E-07
Aromaticity score	8.413E-17	6.953E-21	6.270E-17
Extinction coefficient at 280 nm—All Cys residues appear as half cystines	1.701E-06	3.080E-08	4.875E-08
Extinction coefficient at 280 nm—No Cys residues appear as half cystines	1.864E-06	3.687E-08	5.844E-08
Atomic composition—Carbon	1.967E-25	5.450E-25	5.226E-18
Amino acid composition—F	1.485E-06	9.373E-12	1.123E-08
Amino acid composition—H	7.552E-06	3.296E-07	3.961E-06
Amino acid composition—N	1.161E-08	2.001E-08	4.775E-06
Amino acid composition—P	1.303E-10	5.362E-12	1.515E-08
Amino acid composition—W	7.552E-06	1.842E-06	1.128E-05
Amino acid composition—Y	5.226E-13	1.106E-14	5.057E-13
Number of interactors in the PIA network	7.839E-08	3.209E-06	8.477E-05
Number of interactors in the LEA network	5.942E-04	5.907E-05	3.889E-05
Number of interactors in the MPA network	4.532E-05	3.046E-06	1.371E-04

QFs quantitative features

PIA physical interaction association

LEA literature evidence association

MPA mutant phenotype association

### Quantitative comparison vs. qualitative enrichment analysis

**Figure 5.**
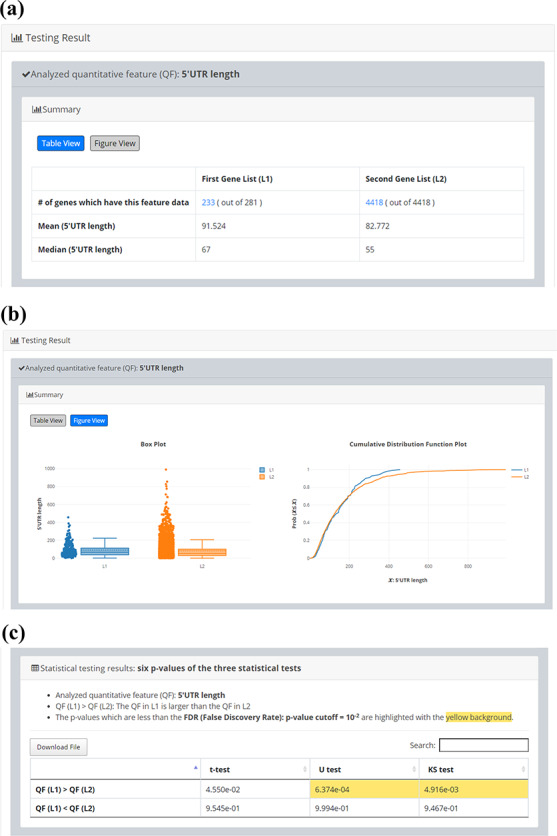
Quantitative comparison of 5’UTR length between 281 iESR genes and the background (4418 genes which have 5’UTR length data). (a) Table View (b) Figure View (c) Statistical testing results. It can be seen that the 5’UTR length of 281 iESR genes (*L1*) is statistically significantly longer than that of the background (*L2*) when using U test or KS test with *p*-value threshold 0.01. Note that only 233 out of 281 iESR genes have 5’UTR length data.

Since quantitative comparison between a single input gene list (*L1*) and the background gene list (*L2*) is possible in YQFC, users may want to correlate the quantitative comparison results with the qualitative enrichment analysis results. The qualitative enrichment analysis is to test the enrichment of a qualitative feature (e.g. GO terms) in the single input gene list compared to the background. Here we provide an example to illustrate the differences between these two testing strategies. Assume that we want to compare the 5ʹUTR length of 281 iESR genes and the background (4418 genes which have 5ʹUTR length data). For quantitative comparison, YQFC found that the 5ʹUTR length of 281 iESR genes (*L1*) is statistically significantly longer than that of the background (*L2*) when using U test or KS test with *P*-value threshold 0.01 (Figure 5). Note that only 233 out of 281 iESR genes have 5ʹUTR length data.

For qualitative enrichment analysis, first we need to define a set of genes with long 5ʹUTR length from the background (4418 genes which have 5ʹUTR length data). Let us define a set of genes with long 5ʹUTR length by including all the genes whose 5ʹUTR lengths are within the largest X% (X = 10, 20, 30, 40) of the background. Then we can use the hypergeometric test to check whether the iESR genes are enriched with genes with long 5ʹUTR length.

If we set the *P*-value threshold for calling enrichment as 0.01, then we can conclude that iESR genes are enriched with genes with long 5ʹUTR length only when X = 40 but not 10, 20 or 30 (Table 4). It can be seen that the testing results of the qualitative enrichment analysis are sensitive to how we define a set of genes with long 5ʹUTR length. Therefore, we suggest researchers to use quantitative comparison rather than qualitative enrichment analysis when quantitative feature data are available.

**Table 4.** For qualitative enrichment analysis, first we need to define a set of genes with long 5′UTR length from the background (4418 genes, which have 5′UTR length data). Let us define a set of genes with long 5′UTR length by including all the genes whose 5′UTR lengths are within the largest X% (X = 10, 20, 30, 40) of the background. Then we can use the hypergeometric test to check whether the iESR genes are enriched with genes with long 5′UTR length. If we set the *P*-value threshold for calling enrichment as 0.01, then we can conclude that iESR genes are enriched with genes with long 5ʹUTR length only when X = 40 but not 10, 20 or 30

X	Expected ratio in background	Observed ratio in iESR genes	*P*-value for enrichment calculated by hypergeometric test
10	10% (441/4418)	12.88% (30/233)	0.08381
20	20% (883/4418)	24.46% (57/233)	0.04971
30	30% (1325/4418)	36.05% (84/233)	0.02398
40	40% (1767/4418)	48.07% (112/233)	0.006232

## Conclusion

In this study, we have developed a web tool called YQFC. We have comprehensively collected 85 quantitative features (including 12 gene features, 4 mRNA features, 52 protein features and 17 network features) in YQFC. For each quantitative feature, YQFC provides three statistical tests (t-test, U test and KS test) to test whether this quantitative feature is statistically different between the two input yeast gene lists. In the case study, we demonstrated the functionality and usefulness of YQFC by identifying 40 distinct quantitative features between rESR genes and iESR genes (Tables 2 and 3). The distinct quantitative features (e.g. 5ʹUTR length, mRNA half-life, transcriptional plasticity, translational efficiency, etc.) identified by YQFC may help researchers to study the underlying molecular mechanisms that differentiate rESR genes from iESR genes. We believe that YQFC is a useful tool to expedite the biological research that uses high-throughput omics technologies. In the future, we will keep updating YQFC once new quantitative features become available in the yeast literature or yeast databases. Now is YQFC Version 1.0. When YQFC has a minor update, we will change the version number to 1.1, 1.2, etc. In the future, if YQFC has a major update, we will change the version number to 2.0.

## Supplementary Material

baaa076_Supp
