# Supplementary material for: YQFC: a web tool to compare quantitative biological features between two yeast gene lists
Source: Database (Oxford). 2020 Nov 11;2020:baaa076. doi: 10.1093/database/baaa076 (PMC7805433; doi:10.1093/database/baaa076)
Supplement: baaa076_Supp [file baaa076_supp.zip › Supplementary Table 1.pdf]

Supplementary Table 1. The detailed sources of each individual feature.

Feature Name	Source	Feature Name	Source	Feature Name	Source
5'UTR length	Nagalakshmi [2]	3'UTR length	Nagalakshmi [2]	Transcription level	Nagalakshmi [2]
Transcriptional frequency	Holstege [1]	mRNA expression level	Holstege [1]	mRNA half-life	Holstege [1]
Transcriptional plasticity	Lin [3]	Translational efficiency	Csárdi [6]	# of mRNA isoforms	Pelechano [4]
Protein half-life	Christiano [5]	Protein abundance in normal/stress conditions[a]	Ho [7]	# of interactors in the (PI, GI) networks	BioGRID [11]
CDS length	SGD [10]	Amino acid composition	SGD [10]	Atomic composition	SGD [10]
Protein physical details (length, molecular weight, pI, aliphatic index, instability index)	SGD [10]	Coding region translation calculation (codon bias, codon adaptation index, frequency of optimal codons, hydropathicity of protein, aromaticity score)	SGD [10]	# of fungal homologs<br># of non-fungal and S. cerevisiae homologs	YeastMine [13]
# of interactors in the (CC, CX, DC, GN, GT, HT, LC, PG, TS) networks	YeastNet [8]	# of GO terms<br># of GO slim terms<br># of pathways<br># of domains<br># of publications<br># of mutant phenotypes	SGD [10]	# of PTMs<br># of transcriptional regulators	YeastMine [13]
# of interactors in the (EPA, FAA, GIA, LEA, MPA, PIA, TFBA, TFRA) networks	YAGM [9]	Extinction coefficients at 280nm (all Cys residues appear as half cystines, no Cys residues appear as half cystines)	SGD [10]	# of interactors in the EPA network	SPELL [12]

[a]Ho *et al.* [7] collected 23 datasets of protein abundance measured in the normal growth condition from the literature. They also collected the datasets of protein abundance measured in 11 different stress conditions (dithiothretiol, H2O2, hydroxyurea, methyl methanesulfonate, etc.) from the literature.

CDS: coding sequence

UTR: untranslated region

GO: gene ontology

PTM: post-translational modification,

pI: isoelectric point,

PI: physical interaction,

GI: genetic interaction,

CC: inferred links by co-citation of two genes across 46,111 pubmed Medline article abstracts for yeast biology from YeastNet,

CX: inferred links by co-expression pattern of two genes (based on high-dimensional gene expression data) from YeastNet,

DC: inferred links by co-occurrence of protein domains between two coding genes from YeastNet

GN: inferred links by similar genomic context of bacterial orthologs of two yeast genes from YeastNet

GT: inferred links by similar profiles of genetic interaction partners from YeastNet

HT: links by high-throughput protein-protein interactions

LC: links by small/medium-scale protein-protein interactions (collected from protein-protein interaction data bases)

PG: Inferred links by similar phylogenetic profiles between two yeast genes from YeastNet

TS: inferred links by 3-D protein structure of interacting orthologous proteins between two yeast proteins from YeastNet

EPA: expression profile association

FAA: functional annotation association

GIA: genetic interaction association

LEA: literature evidence association,

MPA: mutant phenotype association

PIA: physical interaction association

TFBA: transcription factor binding association

TFRA: transcription factor regulation association

References

1. Holstege,F.C., Jennings,E.G., Wyrick,J.J. *et al.* (1998) Dissecting the regulatory circuitry of a eukaryotic genome. *Cell*, **95(5)**, 717-728.
2. Nagalakshmi,U., Wang,Z., Waern,K. *et al.* (2008) The transcriptional landscape of the yeast genome defined by RNA sequencing. *Science*, **320(5881)**, 1344-1349.
3. Lin,Z., Wu,W.S., Liang,H. *et al.* (2010) The spatial distribution of cis regulatory elements in yeast promoters and its implications for transcriptional regulation. *BMC Genomics*, **11**, 581.
4. Pelechano,V., Wei,W. and Steinmetz,L.M. (2013) Extensive transcriptional heterogeneity revealed by isoform profiling. *Nature*, **497(7447)**, 127-131.
5. Christiano,R., Nagaraj,N., Fröhlich,F. *et al.* (2014) Global proteome turnover analyses of the Yeasts S. cerevisiae and S. pombe. *Cell Rep.*, **9(5)**, 1959-1965.
6. Csárdi,G., Franks,A., Choi,D.S. *et al.* (2015) Accounting for experimental noise reveals that mRNA levels, amplified by post-transcriptional processes, largely determine steady-state protein levels in yeast. *PLoS Genet.*, **11(5)**, e1005206.
7. Ho,B., Baryshnikova,A. and Brown,G.W. (2018) Unification of Protein Abundance Datasets Yields a Quantitative Saccharomyces cerevisiae Proteome. *Cell Syst.*, **6(2)**, 192-205.
8. Kim,H., Shin,J., Kim,E. *et al.* (2014) YeastNet v3: a public database of data-specific and integrated functional gene networks for Saccharomyces cerevisiae. *Nucleic Acids Res.*, **42(D1)**, D731-D736.
9. Wu,W.S., Wang,C.C., Jhou,M.J. *et al.* (2015) YAGM: a web tool for mining associated genes in yeast based on diverse biological associations. *BMC Syst Biol.*, **9(Suppl 6)**, S1.
10. Lang,O.W., Nash,R.S., Hellerstedt,S.T. *et al.* (2018) An Introduction to the Saccharomyces Genome Database (SGD). *Methods Mol. Biol.*, **1757**, 21-30.
11. Oughtred,R., Stark,C., Breitkreutz,B.J. *et al.* (2019) The BioGRID interaction database: 2019 update. *Nucleic Acids Res.*, **47(D1)**, D529-D541.
12. Hibbs,M.A., Hess,D.C., Myers,C.L. *et al.* (2007) Exploring the functional landscape of gene expression: directed search of large microarray compendia. *Bioinformatics*, **23(20)**, 2692-2699.

13. Balakrishnan,R., Park,J., Karra,K. *et al.* (2012) YeastMine--an integrated data warehouse for Saccharomyces cerevisiae data as a multipurpose tool-kit. *Database (Oxford)*, **2012**, bar062.
