# Supplementary material for: YQFC: a web tool to compare quantitative biological features between two yeast gene lists
Source: Database (Oxford). 2020 Nov 11;2020:baaa076. doi: 10.1093/database/baaa076 (PMC7805433; doi:10.1093/database/baaa076)
Supplement: baaa076_Supp [file baaa076_supp.zip › Supplementary Table 3.pdf]

Supplementary Table 3. The proportion of genes that contain uORFs in the 5'UTR is much greater for iESR genes than rESR genes. We downloaded the number of uORFs in the 5'UTR of 4149 genes from Lawless et al. [1]. Among 281 iESR genes, only 252 genes have uORFs data. Among 585 rESR genes, only 450 genes have uORFs data. We found that the proportion of genes that contain uORFs in the 5'UTR is statistically greater for iESR genes than rESR genes (*p*-value calculated by the two sample proportion test).

**The proportion of genes that contain uORFs in the 5'UTR of iESR genes (denoted as prop1)**	**The proportion of genes that contain uORFs in the 5'UTR of rESR genes (denoted as prop2)**	***p*-value of claiming prop1> prop2 (calculated by two sample proportion test)**
11.90% (30/252)	4.89% (22/450)	5.677E-04

References

1. Lawless,C., Pearson,R.D., Selley,J.N. *et al.* (2009) Upstream sequence elements direct post-transcriptional regulation of gene expression under stress conditions in yeast. BMC Genomics,**10**, 7.
